# Prognostic Value of MUC2 Expression in Colorectal Cancer: A Systematic Review and Meta-Analysis

**DOI:** 10.1155/2018/6986870

**Published:** 2018-06-05

**Authors:** Chao Li, Didi Zuo, Libin Yin, Yuyang Lin, Chenguang Li, Tao Liu, Lei Wang

**Affiliations:** ^1^Department of Colorectal and Anal Surgery, The First Hospital of Jilin University, Changchun, China; ^2^Department of Endocrinology and Metabolism, The First Hospital of Jilin University, Changchun, China

## Abstract

**Background:**

The reliability of MUC2 as a prognostic marker in colorectal cancer (CRC) is controversial. This study evaluated the association between MUC2 expression levels in CRC tissues and prognosis.

**Methods:**

The PubMed, Web of Science, Embase, Cochrane Library, China Biology Medicine disc (CBMdisc), Wanfang Database, and China National Knowledge Infrastructure (CNKI) databases were searched to identify studies exploring the relationship between MUC2 expression in CRC tissues and overall survival (OS). Pooled hazard ratios (HRs) and risk ratios (RRs) with 95% confidence intervals (CIs) were used to evaluate the associations between MUC2 expression levels and prognosis and MUC2 expression levels and CRC clinicopathological characteristics, respectively.

**Results:**

The meta-analysis included 11 studies (2619 patients). Low MUC2 expression level was significantly associated with poor OS (HR, 1.67; 95% CI, 1.43–1.94; *P* < 0.00001) and disease-free survival (DFS)/recurrence-free survival (RFS) (HR, 1.60; 95% CI, 1.21–2.12; *P* = 0.001) in patients with CRC. Low MUC2 expression level was associated with advanced TNM stage (RR, 1.42; 95% CI, 1.26–1.60; *P* < 0.00001), lymph node metastasis (RR, 1.41; 95% CI, 1.25–1.60; *P* < 0.00001), lymphatic invasion (RR,1.64; 95% CI, 1.26–2.12; *P* = 0.0002), rectal tumor site (RR, 1.26; 95% CI, 1.09–1.46; *P* = 0.001), and large tumor size (RR,1.32; 95% CI, 1.02–1.70; *P* = 0.03). There were no associations between low MUC2 expression level and gender, histological grade, depth of invasion, and distant metastasis.

**Conclusion:**

The low levels of MUC2 in CRC tissues are poor prognostic factor independent of stage or other well-recognized markers of later-stage disease. Large well-designed cohort studies are required to validate MUC2 as a biomarker for poor prognosis in CRC.

## 1. Introduction

Colorectal cancer (CRC) is associated with substantial morbidity and is ranked the third leading cause of cancer-related mortality in the world [1, 2]. The 5-year and 10-year survival rates for CRC are 65% and 58%, respectively [3]. Recurrence is very common in CRC [4, 5], and there is a high risk of subsequent primary cancers in the colon, rectum, and other parts of the digestive system [6]. CRC incidence and mortality rates are rising rapidly in many low- and middle-income countries. The incidence is highest in highly developed countries, but the rates are stabilizing or decreasing in these regions. A 60% increase in the global burden of CRC with more than 2.2 million new cases and 1.1 million deaths is predicted by 2030.

The American Joint Committee on Cancer/Union for International Cancer Control tumor-node-metastasis (TNM) system provides the strongest prognostic parameters for CRC and serves as the basis for treatment decisions [7]. However, the TNM system is less able to predict outcomes in patients with intermediate levels of CRC [8], and there are no definitive biomarkers for monitoring the efficacy of CRC therapies [9]. Therefore, it is necessary to identify new molecular markers that have the potential to predict therapeutic outcomes, serve as therapeutic targets, and improve clinical management in CRC.

Mucins are a family of high molecular weight glycosylated proteins [10] that protect epithelial cells and form the ductal surfaces of several organs [11–13]. To date, approximately 20 mucins have been identified, which can be divided into two subfamilies based on their structure and function, the secreted gel-forming mucins and the transmembrane mucins [14]. Among these, MUC2 is a secreted gel-forming mucin that is encoded within a cluster of genes at the chromosomal locus 11p15 and is thought to share a common ancestor with von Willebrand factor (VWF) [15, 16].

Secreted MUC2 mucin constitutes the major structural component of the mucus in the colon. Colonic mucus has a stratified appearance; the inner mucus layer is attached to the epithelium, is compact, and is devoid of bacteria, while the outer mucus layer is not attached to the epithelium and has an expanded volume due to the action of endogenous proteases, which allows it to be colonized by intestinal bacteria [17]. The inner mucus layer is impervious to bacteria and provides a protective barrier for the colon epithelium. The downregulation of MUC2 expression eliminates this protective mucus barrier, creating a microenvironment in which bacteria can contact the epithelial surface and activate an inflammatory response. Chronic inflammation leads to cellular damage and molecular changes that transform the inflamed epithelium to low-grade dysplasia (LGD), high-grade dysplasia (HGD), and finally CRC [18]. Functionally, MUC2 inhibits the intestinal inflammatory response, thus suppressing the development of intestinal tumors [19, 20]. Conversely, decreased MUC2 expression contributes to CRC by promoting interleukin-6-induced epithelial to mesenchymal transition (EMT), thereby influencing the invasiveness of cancer cells [21, 22]. These mechanisms suggest that MUC2 is an attractive biomarker for diagnosis, immunotherapy, and prognosis in CRC.

Evidence suggests that MUC2 expression is associated with invasion and metastasis in various malignant tumors, including pancreatic cancer [23], gastric carcinoma [24], gallbladder carcinoma [25], extrahepatic bile duct carcinoma [26], breast cancer [27], ovarian cancer [28], ampullary cancer [29], prostate cancer [30], laryngeal cancer [31], and lung cancer [32]. However, the association between MUC2 expression and prognosis in CRC remains to be elucidated. Some studies showed that a low level of MUC2 expression in CRC tissues is associated with poor prognosis [33], while other studies report no obvious correlation [34–37]. Therefore, the objective of the current meta-analysis was to determine the prognostic value of MUC2 in CRC by assessing the association between MUC2 expression levels in CRC tissues and survival. The associations between MUC2 expression levels and several CRC clinicopathological characteristics were also investigated.

## 2. Materials and Methods

### 2.1. Search Strategy

Two reviewers (Chao Li, Didi Zuo) independently searched the PubMed, Web of Science, Embase, Cochrane Library, China Biology Medicine disc (CBMdisc), Wanfang Database, and China National Knowledge Infrastructure (CNKI) databases from inception through November 9, 2017, using the following MeSH terms and free-text words: “colorectal neoplasms”/“colorectal cancer”/“colon cancer”/“rectal cancer” and “mucin 2”/“MUC2” and “survival”/“outcome”/“prognosis”/“mortality”. A manual search of the reference lists of relevant articles was conducted to identify additional relevant studies. The search was limited to articles published in the English or Chinese language.

### 2.2. Inclusion and Exclusion Criteria

Inclusion criteria were as follows: (1) study design: cohort, (2) population: patients with CRC, (3) parameter: MUC2 expression levels in CRC tissue samples, and (4) outcomes: associations between MUC2 expression levels in CRC tissues and overall survival (OS). Exclusion criteria were as follows: (1) duplicate publications; (2) in vitro or animal studies; (3) conference reports, reviews, books, case reports, or letters; or (4) insufficient data. When articles reported data from the same study, data from the most recent article was included.

### 2.3. Study Selection and Data Extraction

Two reviewers (Chao Li, Didi Zuo) independently examined titles and abstracts to select eligible studies. The full text of potentially relevant studies was retrieved and examined to determine which studies met the inclusion criteria.

Two reviewers (Chao Li, Didi Zuo) independently extracted data from eligible studies including first author's last name, year of publication, country, number of patients, mean age of patients, time of follow-up, MUC2 detection method, MUC2 antibody, cutoff values used to assess MUC2 expression levels, and clinical outcomes. Disagreements about study selection and data extraction were resolved by discussion with a third reviewer (Libin Yin) until consensus was reached.

### 2.4. Methodological Quality

Two reviewers (Chao Li, Didi Zuo) independently assessed the methodological quality of the included studies using the modified Newcastle-Ottawa Scale (NOS) [38], which allocates a maximum of 9 points according to the quality of the selection, comparability, and outcomes of the study populations. Study quality was defined as poor (0–3), fair (4–6), or good (7–9). Publication bias was assessed using Begg's rank correlation test and Egger's linear regression [39].

Disagreements about the assessment of methodological quality were resolved by discussion with a third reviewer (Libin Yin) until consensus was reached.

### 2.5. Statistical Analysis

Statistical analyses were performed using Review Manager, version 5.3 (Cochrane Collaboration, Copenhagen, Denmark), and STATA, version 12.0 (Stata Corporation, College Station, TX, USA). Hazard ratios (HRs) with 95% confidence intervals (CIs) were used to evaluate the association between MUC2 expression levels (low versus high) in CRC tissues and OS. HR data were obtained directly from studies or were calculated from Kaplan-Meier curves using Engauge Digitizer, version 4.1 (http://markummitchell.github.io/engauge-digitizer/) [40]. An HR > 1 suggested a worse prognosis in CRC patients with a low level of MUC2 expression, and an HR < 1 indicated a better prognosis. Risk ratios (RRs) with 95% CIs were used to evaluate the associations between MUC2 expression levels (low versus high) in CRC tissues and CRC clinicopathological characteristics, including TNM stage, lymph node metastasis, lymphatic invasion, tumor site, tumor size, gender, histological grade, depth of invasion, and distant metastasis. An RR > 1 suggested that a clinicopathological characteristic was associated with a low level of MUC2 expression, and an RR < 1 indicated a characteristic was associated with a high level of MUC2 expression. A random-effects model was used to pool studies with significant heterogeneity, as determined by the chi-squared test (*P* ≤ 0.10) and the inconsistency index (*I* ^2^ ≥ 50%) [41, 42]. Sources of heterogeneity were explored using metaregression. Sensitivity analysis omitting one study at a time was conducted to investigate the robustness of the findings. *P* < 0.05 was considered statistically significant.

## 3. Results

### 3.1. Search Results

**Figure 1:**
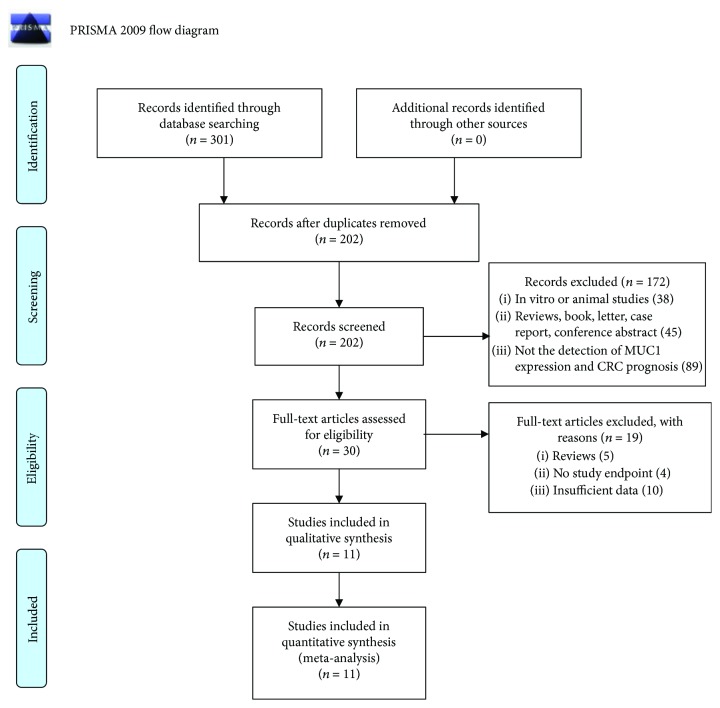
Flow chart of literature search. From: Moher D, Libertati A, Tetzlaff J, Altman DG, The PRISMA Group (2009). Preferred Reporting Items for Systematic Reviews and Meta-Analyses: The PRISMA Statement. PloS Med 6(7): e1000097. doi:10.1371/journal.pmed1000097. For more information, visit http://www.prisma-statement.org.

The searches identified 301 articles. Titles and abstracts were screened, and 99 duplicates and 172 studies that did not meet the inclusion criteria were excluded. The full text of 30 articles was retrieved for further review. Of these, 5 review articles, 4 studies that did not report an endpoint, and 10 studies with insufficient data were excluded. Finally, 11 studies [37, 43–52] were found eligible for inclusion in our review (Figure 1).

### 3.2. Characteristics of the Included Studies

The characteristics of the included studies are shown in Table 1. The 11 eligible studies [37, 43–52] were published between 2007 and 2017. Overall, the studies included 2619 patients (range, 35–938 patients). The mean age of patients ranged from 52.9 to 70.5 years, and the median follow-up ranged from 36 to 128 months. Various anti-MUC2 monoclonal antibodies were utilized, including Ccp-58 MRQ-18, NCL-MUC2, and H300. All studies quantified MUC2 expression levels in CRC tissues by immunohistochemistry (IHC). The measurements in all of the included studies were of overall intensity of staining or of percentage cells that were stained. However, each study used a different cutoff point.

### 3.3. Methodological Quality

The methodological quality of all included studies was good (NOS score > 7) (Table 2). Begg's rank correlation test and Egger's linear regression revealed no publication bias (Begg's test: OS, *P* = 0.152; DFS/RFS, *P* = 0.806; TNM stage, *P* = 0.711; lymph node metastasis, *P* = 0.536; lymphatic invasion, *P* = 1.000; tumor site, *P* = 1.000; tumor size, *P* = 1.000; gender, *P* = 0.060, histological grade, *P* = 0.707; depth of invasion, *P* = 0.707; and distant metastasis, *P* = 1.000) (Supplementary File 1).

### 3.4. Outcomes

#### 3.4.1. MUC2 Expression and Overall Survival in CRC

**Figure 2:**
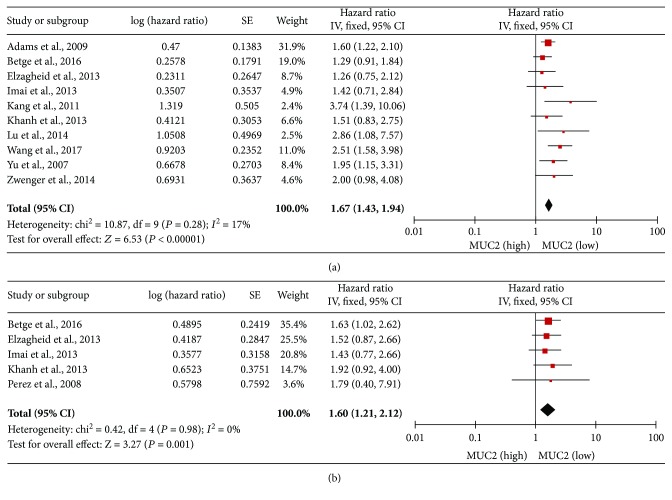
Associations between the MUC2 expression level and OS (a) and DFS/RFS (b) in CRC.

The association between the MUC2 expression level in CRC tissues and OS was investigated in 10 studies. The meta-analysis demonstrated that a low level of MUC2 expression was associated with poor OS in patients with CRC (HR, 1.67; 95% CI, 1.43–1.94; *P* < 0.00001; Figure 2(a)). There was no evidence of significant heterogeneity between the studies (*P* = 0.28, *I* ^2^ = 17%).

#### 3.4.2. MUC2 Expression and Disease-Free Survival/Recurrence-Free Survival

The association between the MUC2 expression level in CRC tissues and DFS/RFS was investigated in 5 studies. The meta-analysis demonstrated that a low level of MUC2 expression was associated with shorter DFS/RFS in patients with CRC (HR, 1.60; 95% CI, 1.21–2.12; *P* = 0.001; Figure 2(b)). There was no evidence of significant heterogeneity between the studies (*P* = 0.98, *I* ^2^ = 0%).

#### 3.4.3. MUC2 Expression and TNM Stage

**Figure 3:**
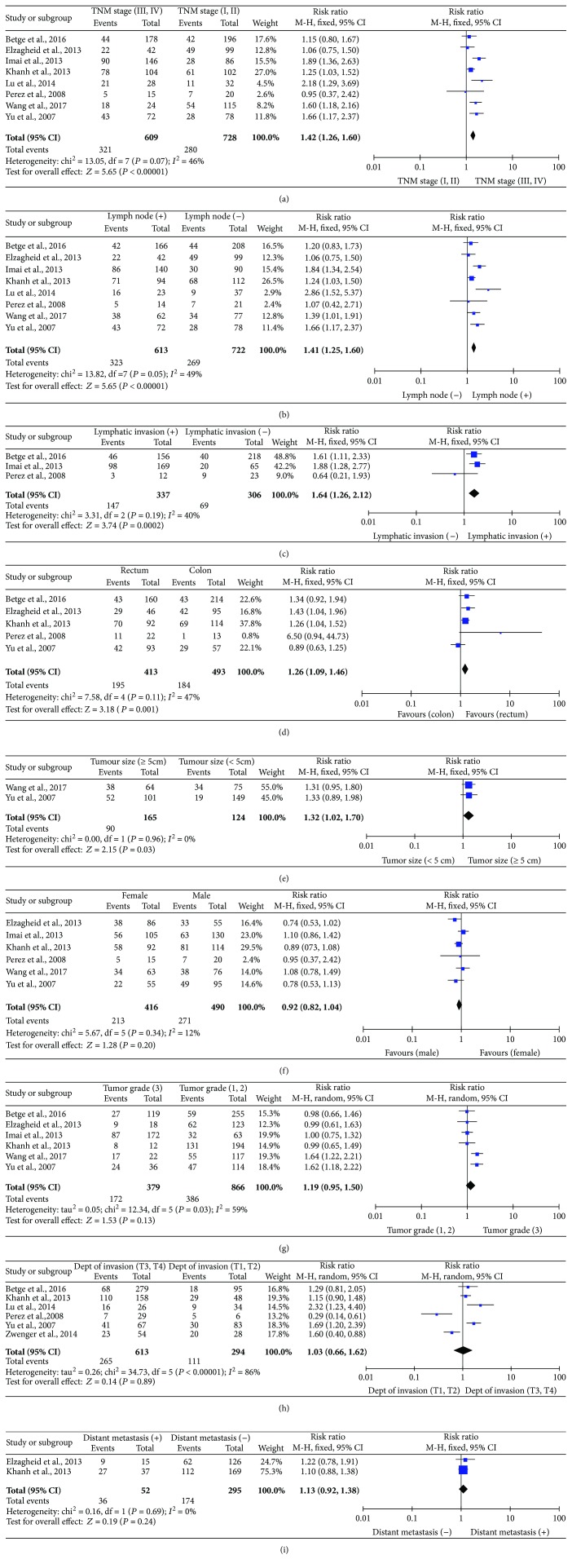
Associations between the MUC2 expression level and CRC clinicopathological characteristics. (a) TNM stage, (b) lymph node metastasis, (c) lymphatic invasion, (d) tumor site, (e) tumor size, (f) gender, (g) histological grade, (h) depth of invasion, (i) distant metastasis.

The association between the MUC2 expression level in CRC tissues and TNM stage was investigated in 8 studies. The meta-analysis demonstrated that a low level of MUC2 expression was associated with CRC in the advanced stages (TNM stage III/IV) compared to the localized stages (TNM stage I/II) (RR, 1.42; 95% CI, 1.26–1.60; *P* < 0.00001; Figure 3(a)). There was no evidence of significant heterogeneity between the studies (*P* = 0.007, *I* ^2^ = 46%).

#### 3.4.4. MUC2 Expression and Lymph Node Metastasis

The association between the MUC2 expression level in CRC tissues and lymph node metastasis was investigated in 8 studies. The meta-analysis demonstrated that a low level of MUC2 expression was associated with lymph node metastasis in patients with CRC (RR, 1.41; 95% CI, 1.25–1.60; *P* < 0.00001; Figure 3(b)). There was no evidence of significant heterogeneity between the studies (*P* < 0.00001, *I* ^2^ = 49%).

#### 3.4.5. MUC2 Expression and Lymphatic Invasion

The association between the MUC2 expression level in CRC tissues and lymphatic invasion was investigated in 3 studies. The meta-analysis demonstrated that a low level of MUC2 expression was associated with lymphatic invasion in patients with CRC (RR, 1.64; 95% CI, 1.26–2.12; *P* = 0.0002; Figure 3(c)). There was no evidence of significant heterogeneity between the studies (*P* = 0.19, *I* ^2^ = 40%).

#### 3.4.6. MUC2 Expression and Tumor Site

The association between the MUC2 expression level in CRC tissues and tumor site was investigated in 5 studies. The meta-analysis demonstrated that a low level of MUC2 expression was associated with CRC in the rectum compared to the colon (RR, 1.26; 95% CI, 1.09–1.46; *P* = 0.001; Figure 3(d)). There was no evidence of significant heterogeneity between the studies (*P* = 0.11, *I* ^2^ = 47%).

#### 3.4.7. MUC2 Expression and Tumor Size

The association between the MUC2 expression level in CRC tissues and tumor size was investigated in 2 studies. The meta-analysis demonstrated that a low level of MUC2 expression was associated with large tumors compared to small tumors in patients with CRC (RR, 1.32; 95% CI, 1.02–1.70; *P* = 0.03; Figure 3(e)). There was no evidence of heterogeneity between the studies (*P* = 0.96, *I* ^2^ = 0%).

#### 3.4.8. MUC2 Expression and Other Clinical Features

The associations between the MUC2 expression level in CRC tissues and other clinicopathological characteristics were investigated The meta-analysis demonstrated that a low level of MUC2 expression did not show an association with gender (female versus male: RR, 0.92; 95% CI, 0.82–1.04; *P* = 0.20; Figure 3(f)), histological grade (RR 1.19; 95% CI, 0.95–1.50; *P* = 0.13; Figure 3(g)), depth of invasion (T3, T4 versus T1, T2: RR, 1.03; 95% CI, 0.66–1.62; *P* = 0.89; Figure 3(h)), and distant metastasis (positive versus negative: RR, 1.13;95% CI, 0.92–1.38; *P* = 0.24; Figure 3(i)).

### 3.5. Sensitivity Analysis

Sensitivity analysis omitting one study at a time indicated that the findings of this meta-analysis were robust (Supplementary File 2).

### 3.6. Metaregression

The metaregression of factors influencing the association of MUC2 expression with OS and DFS/RFS in CRC was performed. None of the covariates analyzed, including year, country, antibody, or cutoff values, influenced the association (Supplementary File 3).

## 4. Discussion

Evidence suggests that CRC tissues express low levels of MUC2 and that MUC2 plays a role in the development and progression of CRC. However, the prognostic value of MUC2 in CRC remains to be elucidated. Although a previous meta-analysis [53] investigated the association between MUC2 expression and CRC clinicopathological characteristics, to the authors' knowledge, the current study is the first meta-analysis to evaluate the prognostic value of MUC2 expression in CRC. The results showed that a low level of MUC2 expression in CRC tissues was associated with poor OS and DFS/RFS. These findings suggest that MUC2 has a protective role in CRC, which may be explained by several mechanisms. MUC2 silencing may promote CRC metastasis by interleukin-6-induced EMT, which contributes to the invasiveness of cancer cells [21, 54]. MUC2 downregulation may contribute to chronic inflammation [55], generating a microenvironment that results in genomic instability [56]. In addition, MUC2 downregulation has been associated with increased expression of tumor-associated antigen carcinoembryonic antigen-related cell adhesion molecules 5 and 6 (CEACAM5 and CEACAM6), which are involved in cell adhesion, migration, tumor invasion, and metastasis [57, 58]. Taken together, these data indicate that MUC2 may serve as a therapeutic target with potential to improve clinical management in CRC and suggest that randomized controlled clinical trials investigating the role of MUC2 in CRC therapy are warranted.

In accordance with our findings, previous studies indicated that a low level of MUC2 expression in CRC tissues is an indicator of poor prognosis. Betge et al. [48], showed that loss of MUC2 expression in CRC tissues was a predictor of adverse outcome. Wang et al. [45] reported that low MUC2 expression in CRC tissues was significantly associated with lymph node metastasis, poor cellular differentiation, and an advanced tumor stage in CRC, and patients with high MUC2 expression in CRC tissues had higher 5-year survival than patients with low MUC2 expression. Lugli et al. [59] found that the loss of MUC2 in CRC tissues was an adverse prognostic factor for survival in mismatch-repair- (MMR-) proficient and MLH1-negative CRC. In contrast, other studies showed a lower 3-year survival rate in patients with high MUC2 expression in CRC tissues compared to low MUC2 expression (0% versus 60%, resp.) [60]. Espinoza et al. [34] reported that MUC2 expression levels in CRC tissues did not significantly correlate with DFS among African Americans and Caucasian Americans.

As CRC clinicopathological characteristics are often used in clinical practice to predict prognosis, the current study comprehensively explored the association between MUC2 expression levels in CRC tissues and CRC clinicopathological characteristics. The results showed that a low level of MUC2 expression was associated with advanced TNM stage, lymph node metastasis, lymphatic invasion, tumor in the rectum versus the colon, and large tumor size. However, there were no associations between MUC2 expression level and gender, histological grade, depth of invasion, and distant metastasis. Previous reports have demonstrated that advanced TNM stage, lymph node metastasis, lymphatic invasion, rectal tumor site, and large tumor size are predictors of poor prognosis in CRC [44, 46, 61–63]. These data, together with the findings from the current study, imply that the low levels of MUC2 expression in CRC tissues may be used as a biomarker for poor prognosis.

As MUC2 expression levels in CRC tissues are important for diagnosis and prognosis in CRC, MUC2 levels in CRC tissues may be used to guide clinical decision-making. MUC2 may be detected by immunohistochemistry, which is a relatively simple and cost-effective method that could gain widespread acceptance. However, as MUC2 is determined postoperatively in CRC tissue samples, continuous monitoring of MUC2 expression levels throughout the course of disease and with treatment will be challenging.

This study was associated with some limitations. First, some of the included studies did not directly report HRs; instead, they had to be extracted from Kaplan-Meier curves, which may have affected the robustness of our results. Second, the potential sources of heterogeneity between the studies included publication year, country, MUC2 antibody, and cutoff values for MUC2 expression; however, the metaregression analysis revealed that none of these factors were significant sources of heterogeneity. Last, the sample size in the study was small; therefore, the findings should be considered preliminary.

In conclusion, the current study suggests that a low level of MUC2 expression is an independent factor of poor prognosis in colorectal cancer and also associated with later TNM stage, presence of lymph node metastasis, rectal tumor site, and large tumor size. However, the clinical relevance of MUC2 downregulation in CRC tissues remains to be elucidated. Large well-designed cohort studies are required to validate MUC2 as a biomarker for poor prognosis in CRC.

## Figures and Tables

**Table 1:** Main characteristics of the included publications.

Publication	Year	Country	Patient number	Gender	Antibody	Cutoff (low/high level)	Method	Outcome	TNM stage	Mean age (years)	Median follow-up (months)	NOS score
Adams et al.	2009	Switzerland	938	422/510	NR	High (>5%)	IHC	OS	I–IV	70.5	128	7
Betge et al.	2016	Germany	381	215/166	Ccp-58	High (>0%)	IHC	OS/DFS	I–IV	68.5	NR	8
Elzagheid et al.	2013	Libya	141	55/86	MRQ-18	High (>0%)	IHC	OS/DFS	I–IV	NR	77	8
Imai et al.	2013	Japan	250	136/114	Ccp-58	High (≥25%)	IHC	OS/RFS	I–IV	NR	NR	8
Kang et al.	2011	Korea	229	NR	NR	High (staining score ≥ 6)	IHC	OS	II-III	NR	108	7
Khanh et al.	2013	Japan	206	114/92	Ccp-58	High (≥5%)	IHC	OS/RFS	I–IV	NR	NR	8
Lu et al.	2014	China	60	33/27	Ccp-58	High (>5%)	IHC	OS	I–IV	52.9	NR	8
Perez et al.	2008	Brazil	35	20/15	Ccp-58	High (>10%)	IHC	OS/DFS	I–IV	62.2	NR	7
Wang et al.	2017	China	139	76/63	NCL-MUC2	High (>20%)	IHC	OS	II–IV	NR	NR	8
Yu et al.	2007	China	150	95/55	Ccp-58	High (staining score ≥ 2)	IHC	OS	I–IV	57.5	NR	8
Zwenger et al.	2014	Argentina	90	52/38	H300	High (staining score > 0)	IHC	OS	I–IV	NR	NR	8

IHC: immunohistochemistry; OS: overall survival; DFS: disease-free survival; RFS: recurrence-free survival; NR: not reported; NOS score: Newcastle-Ottawa Scale score.

**Table 2:** Quality assessment of the included studies.

First author, year	Selection^1^				Comparability^2^	Outcome^3^			
	Representativeness of exposed cohort ★	Selection of nonexposed cohort ★	Ascertainment of exposure ★	No primary outcome was present at the start of study ★	Comparable on confounder ★★	Outcome assessment ★	Adequate follow-up ★	Loss to follow-up ★	Total score
Adams et al., 2009	★	★	★		★★	★	★		7
Betge et al., 2016	★	★	★	★	★	★	★	★	8
Elzagheid et al., 2013	★	★	★		★★	★	★	★	8
Imai et al., 2013	★	★	★		★★	★	★	★	8
Kang et al., 2011	★	★	★	★	★	★	★		7
Khanh et al., 2013	★	★	★		★★	★	★	★	8
Lu et al., 2014	★	★	★		★★	★	★	★	8
Perez et al., 2008	★	★	★	★	★	★	★		7
Wang et al., 2017	★	★	★		★★	★	★	★	8
Yu et al., 2007	★	★	★		★★	★	★	★	8
Zwenger et al., 2014	★	★	★		★★	★	★	★	8

^1^“Selection” part includes representativeness of cases, selection of controls, exposure ascertainment, and no death when investigation begins. ^2^“Comparability” part includes comparable on confounders. ^3^“Outcome” part includes outcome assessment, adequate follow-up, and loss to follow-up rate. ★ represents score of 1. ★★ represents score of 2.

## Supplementary Materials

Supplementary 1 Supplementary File 1: publication bias. (a) OS, (b) DFS/RFS, (c) TNM stage, (d) lymph node metastasis, (e) lymphatic invasion, (f) tumor site, (g) tumor size, (h) gender, (i) tumor grade, (j) depth of invasion, (k) distant.

Supplementary 2 Supplementary File 2: sensitivity analysis. (a) OS, (b) DFS/RFS, (c) TNM stage, (d) lymph node metastasis, (e) lymphatic invasion, (f) tumor site, (g) tumor size, (h) gender, (i) tumor grade, (j) depth of invasion, (k) distant.

Supplementary 3 Supplementary File 3: results of metaregression analysis exploring the source of heterogeneity with OS and DFS.
