# Supplementary figures and images for: Prognostic Value of MUC2 Expression in Colorectal Cancer: A Systematic Review and Meta-Analysis
Source: Gastroenterol Res Pract. 2018 Jun 5;2018:6986870. doi: 10.1155/2018/6986870 (PMC6008766; doi:10.1155/2018/6986870)

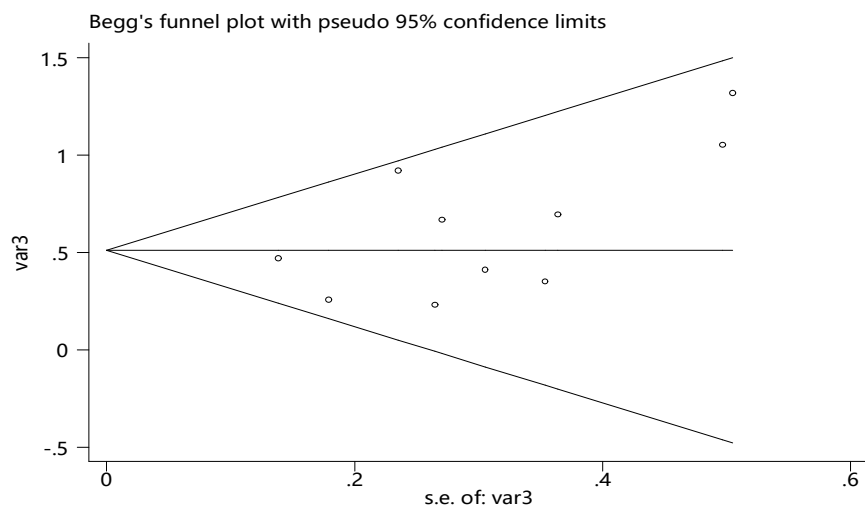

(a)

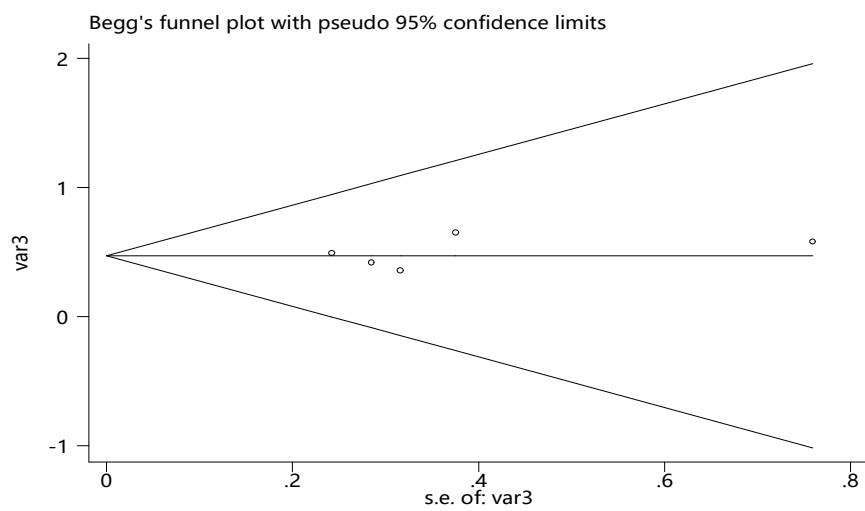

(b)

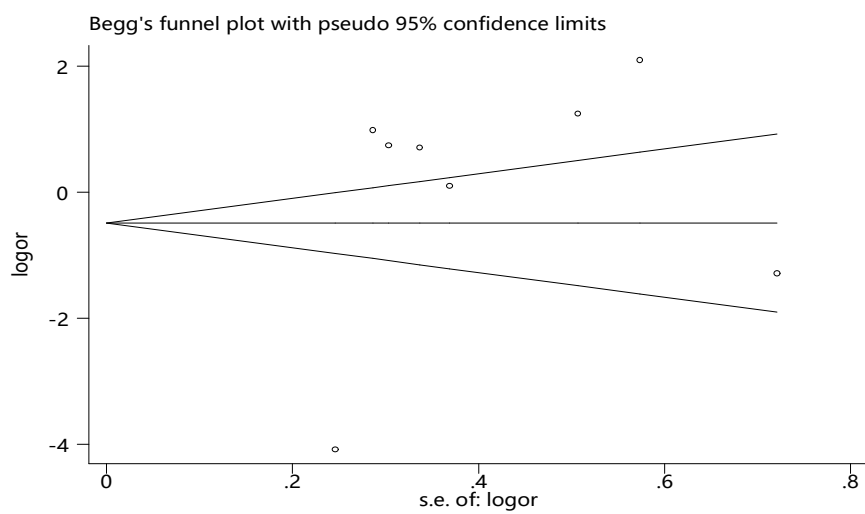

(c)

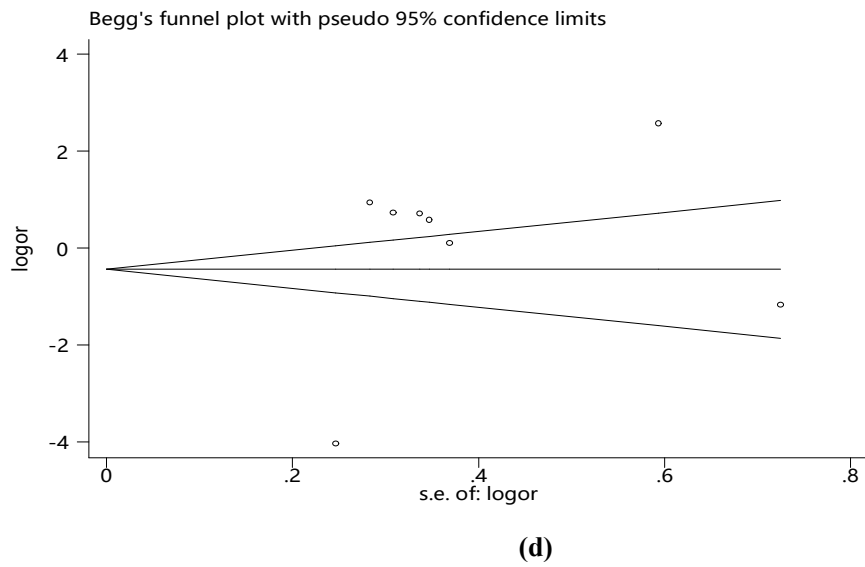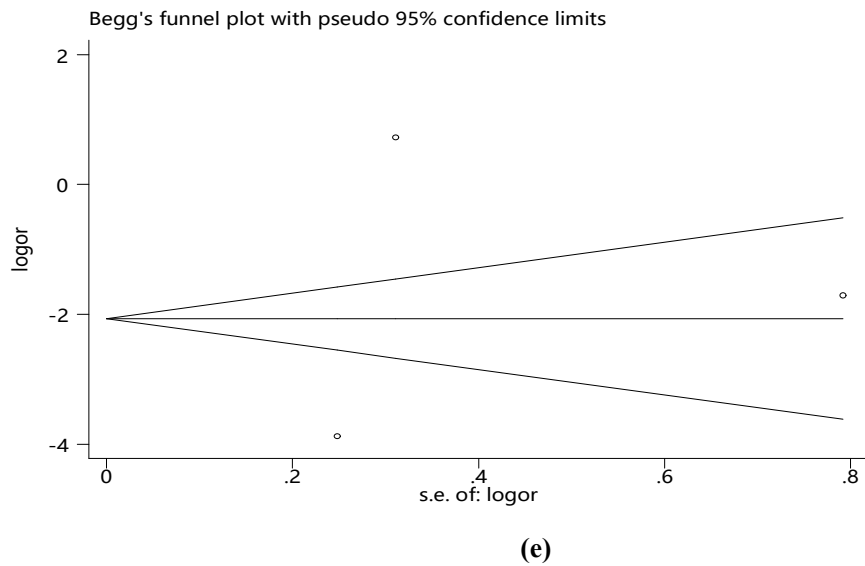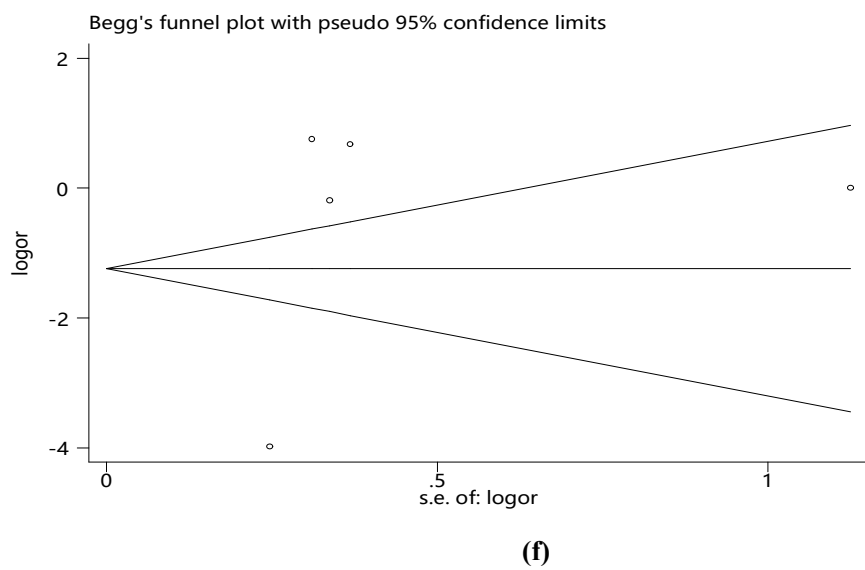

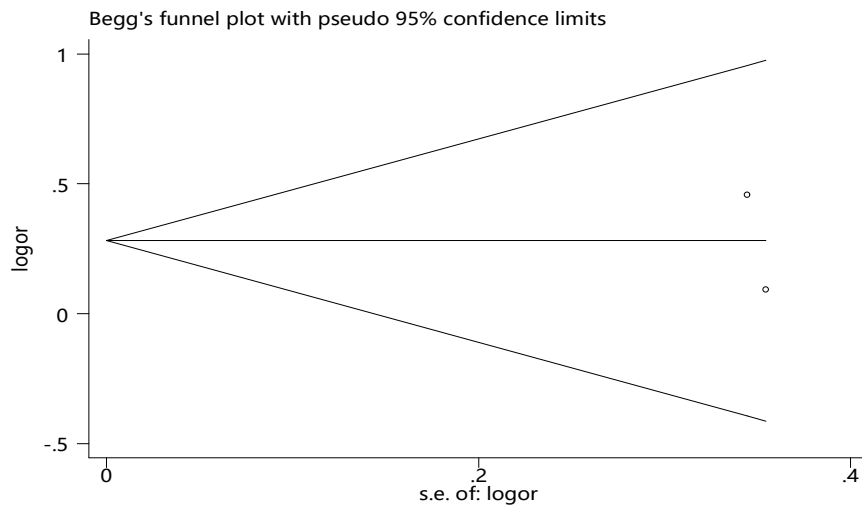

(g)

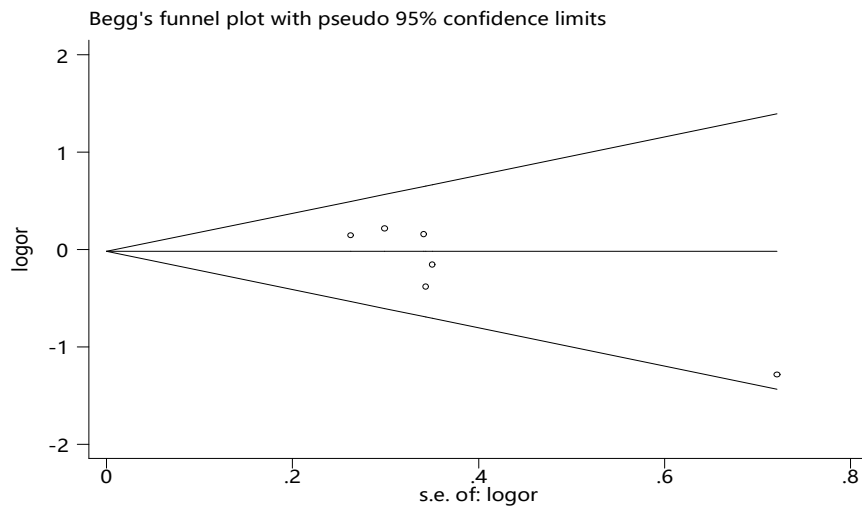

(h)

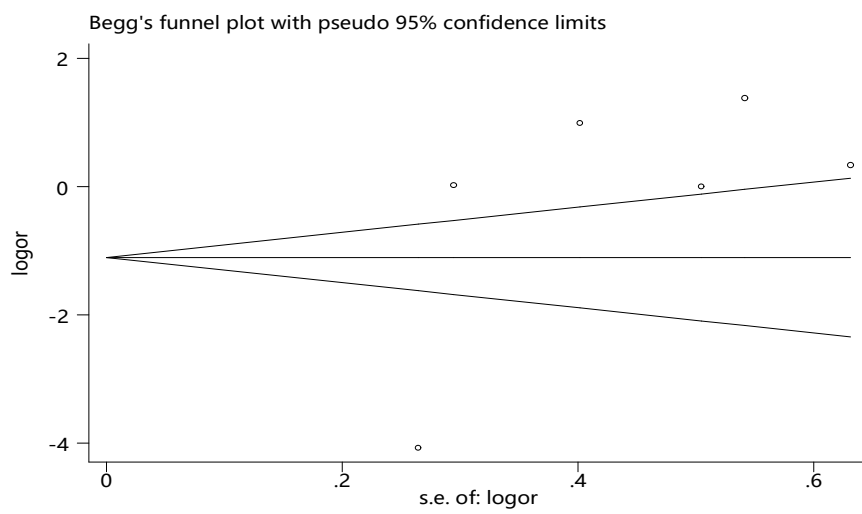

(i)

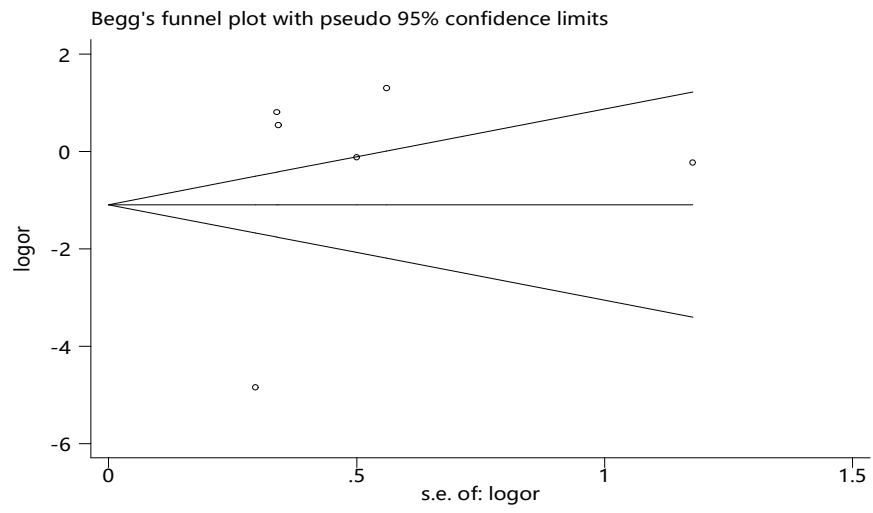

(j)

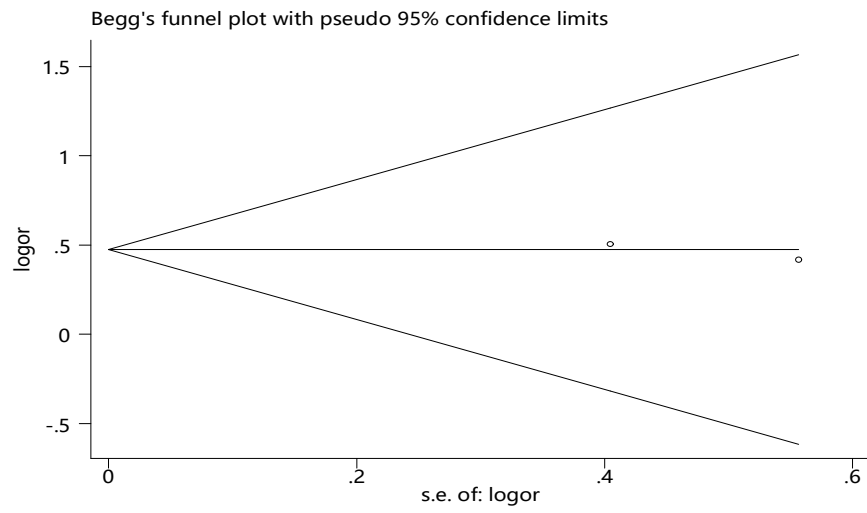

(k)

Supplement: Supplementary 1 — Supplementary File 1: publication bias. (a) OS, (b) DFS/RFS, (c) TNM stage, (d) lymph node metastasis, (e) lymphatic invasion, (f) tumor site, (g) tumor size, (h) gender, (i) tumor grade, (j) depth of invasion, (k) distant. [file 6986870.f1.pdf]

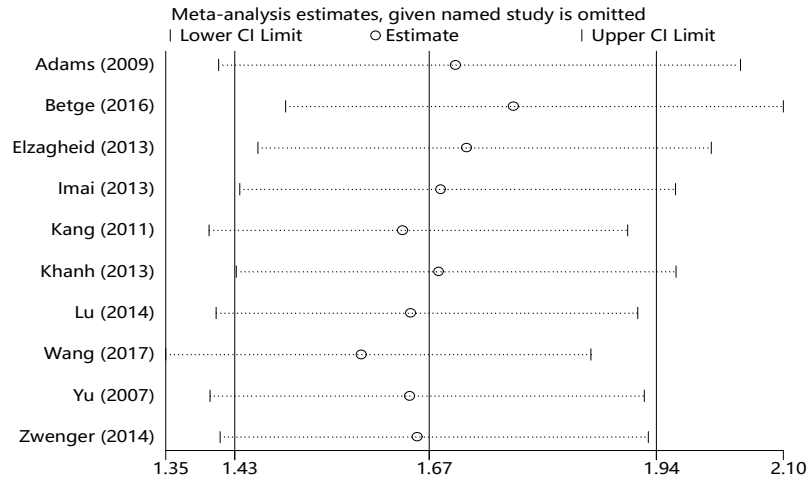

(a)

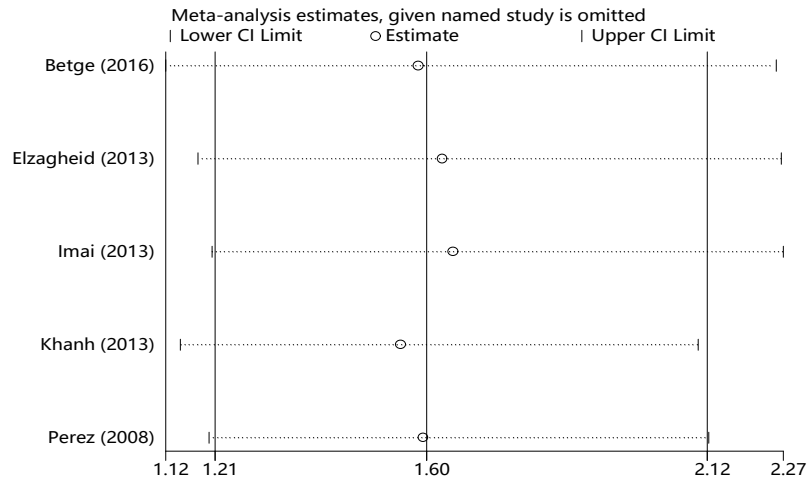

(b)

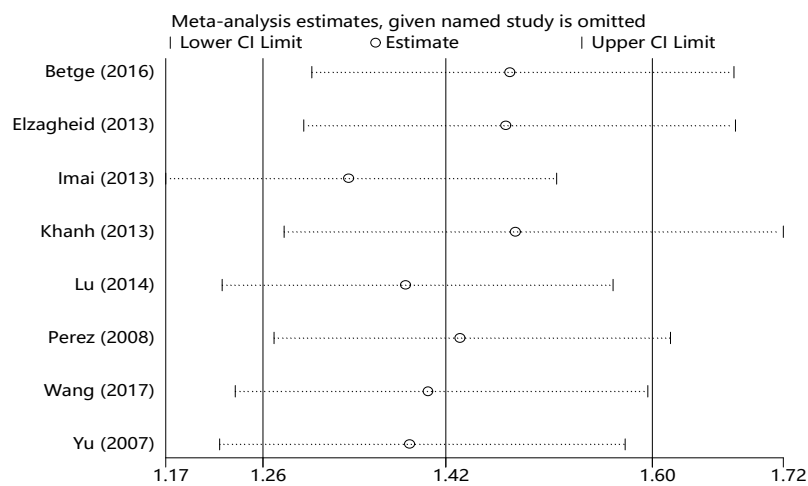

(c)

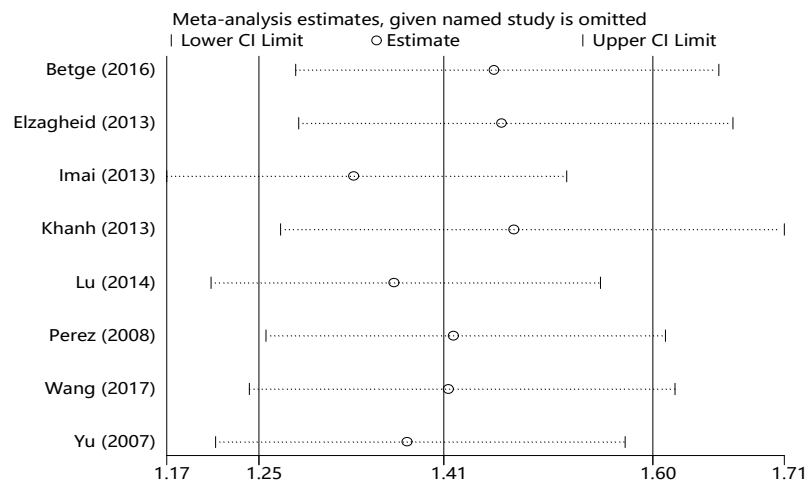

(d)

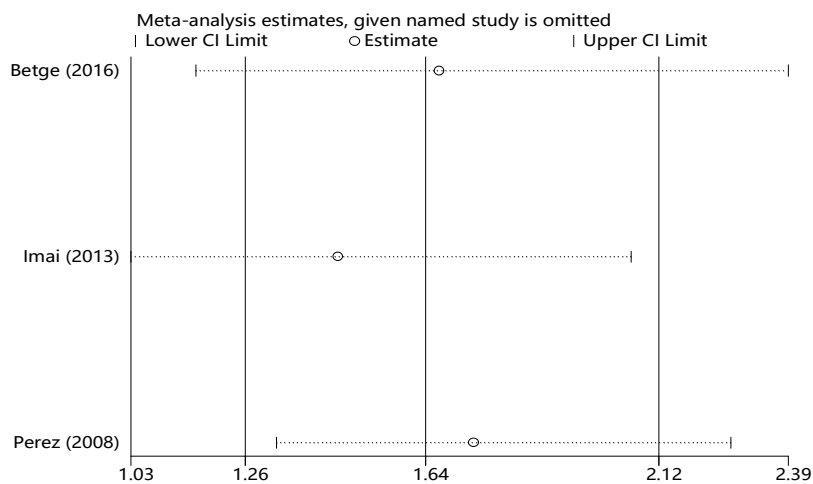

(e)

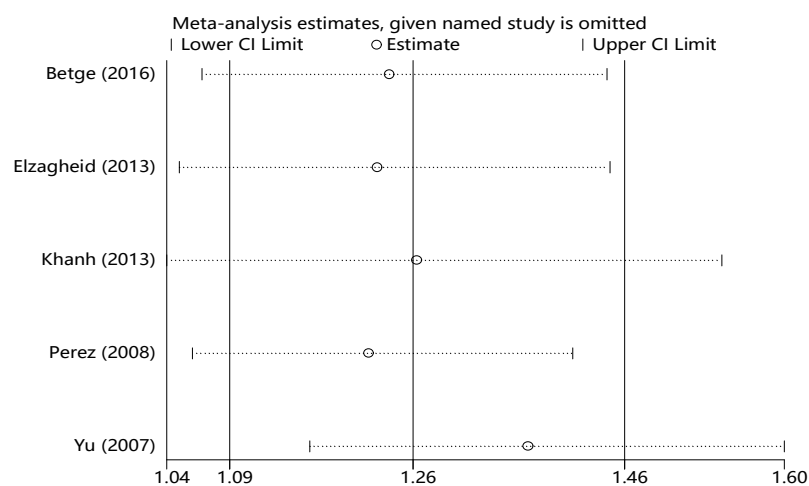

(f)

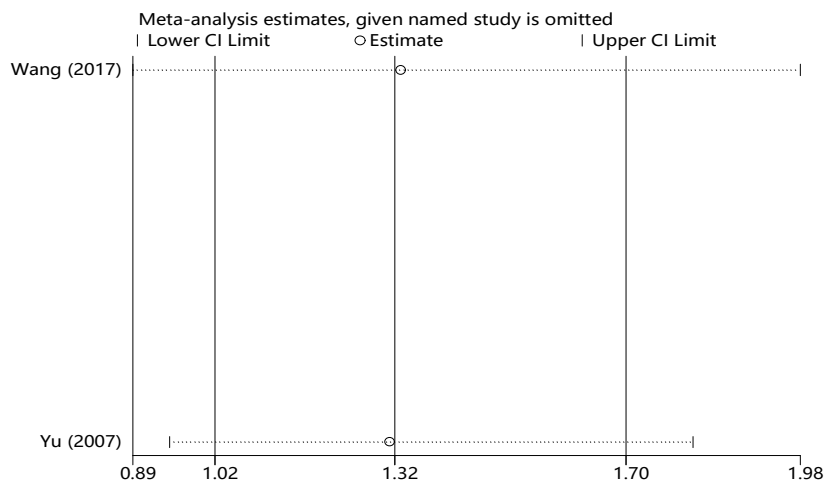

(g)

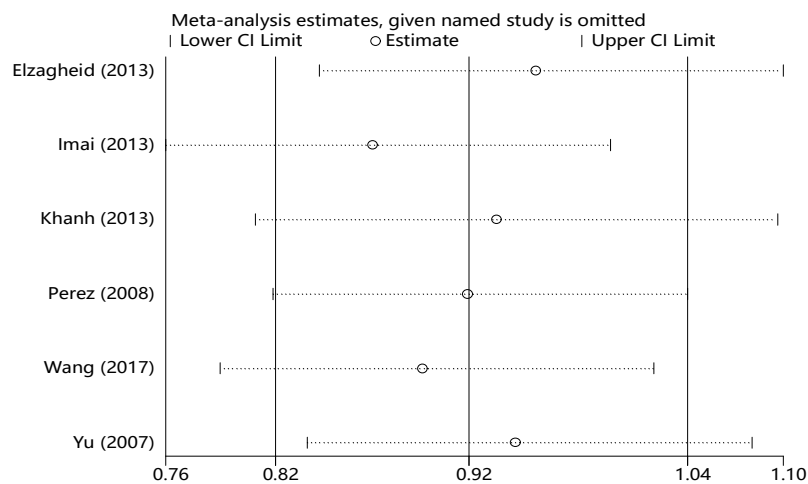

(h)

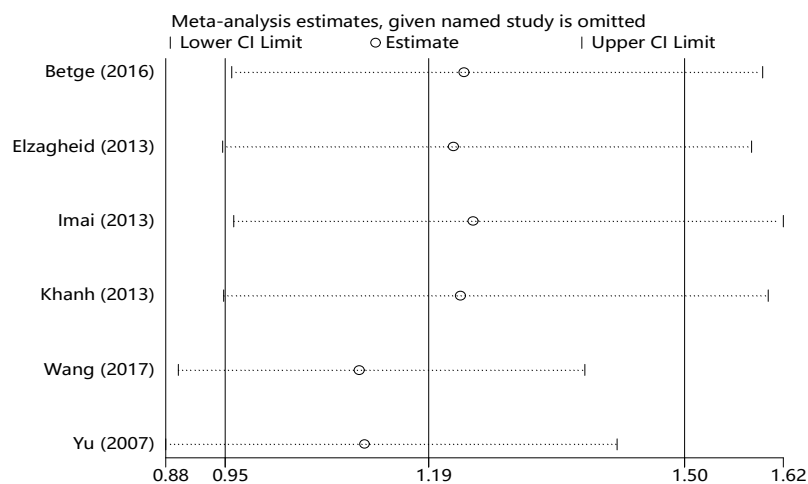

(i)

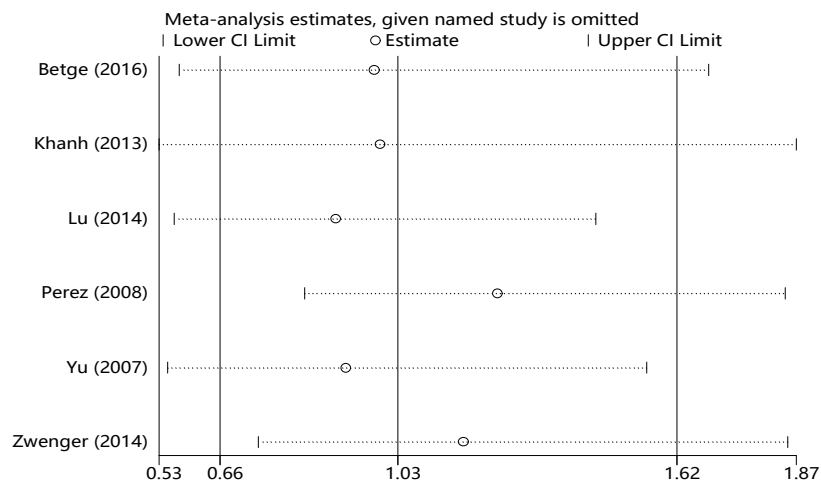

(j)

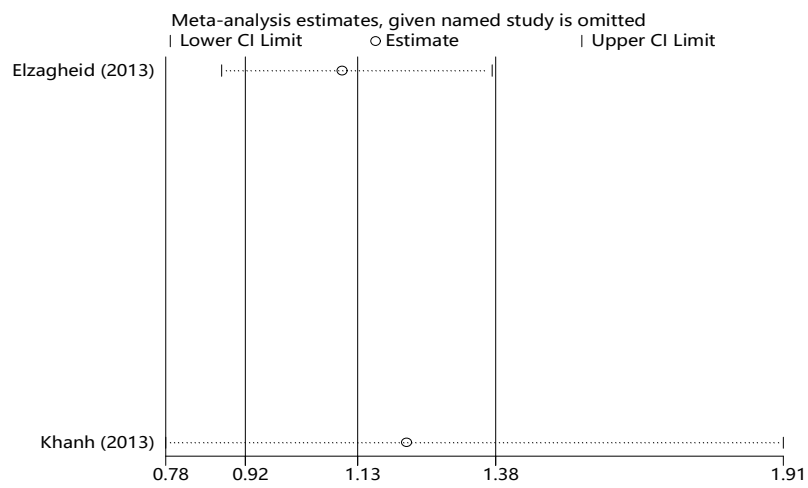

(k)

Supplement: Supplementary 2 — Supplementary File 2: sensitivity analysis. (a) OS, (b) DFS/RFS, (c) TNM stage, (d) lymph node metastasis, (e) lymphatic invasion, (f) tumor site, (g) tumor size, (h) gender, (i) tumor grade, (j) depth of invasion, (k) distant. [file 6986870.f2.pdf]
