# Supplementary material for: Prognostic Value of MUC2 Expression in Colorectal Cancer: A Systematic Review and Meta-Analysis
Source: Gastroenterol Res Pract. 2018 Jun 5;2018:6986870. doi: 10.1155/2018/6986870 (PMC6008766; doi:10.1155/2018/6986870)
Supplement: Supplementary 3 — Supplementary File 3: results of metaregression analysis exploring the source of heterogeneity with OS and DFS. [file 6986870.f3.doc]

Supplementary Table . Results of meta-regression analysis exploring the source of heterogeneity with OS and DFS.

| Covariates | Multivariable analysis(OS) | | | | Multivariable analysis(DFS) | | |
| --- | --- | --- | --- | --- | --- | --- | --- |
|  | Coefficient | SE | P value | Coefficient | | SE | P value |
| Year | 0.016 | 0.028 | 0.584 | -0.020 | | 0.159 | 0.921 |
| Antibody | 0.173 | 0.122 | 0.218 | -0.131 | | 0.607 | 0.865 |
| Cut-off | 0.067 | 0.120 | 0.603 | 0.070 | | 0.645 | 0.932 |
| Country | -0.486 | 0.196 | 0.056 | N | | N | N |

N=From the non-Chinese population
